# Influence of statin intervention on peripheral neuropathy in patients treated with anticancer drugs identified from the insurer database

**DOI:** 10.1186/s40780-025-00428-3

**Published:** 2025-04-07

**Authors:** Fuka Aizawa, Kenta Yagi, Maki Sato, Takahiro Niimura, Mitsuhiro Goda, Yuki Izawa-Ishizawa, Keisuke Ishizawa

**Affiliations:** 1 Department of Pharmacy, Tokushima University Hospital, Tokushima, Japan; 2 Department of Clinical Pharmacology and Therapeutics, Graduate School of Biomedical Sciences, Tokushima University, Tokushima, Japan; 3 Clinical Research Centre for Developmental Therapeutics, Tokushima University Hospital, Tokushima, Japan; 4 Department of General Medicine, Taoka Hospital, Tokushima, Japan

**Keywords:** HMG-CoA reductase inhibitor, Chemotherapy-induced peripheral neuropathy, Oxaliplatin, Paclitaxel, Supportive care

## Abstract

**Background:**

Statins, hydroxymethylglutaryl-CoA reductase inhibitors, possess neuroprotective properties. Given the potential neuroprotective properties of statins and their prevalent use in clinical settings, we aimed to investigate their impact on chemotherapy-induced peripheral neuropathy (CIPN) in Japan by assessing both their safety and efficacy in this context.

**Methods:**

We conducted a retrospective observational study using the Japan Medical Data Centre database, which includes data from 2005 to 2021. We included patients who underwent anticancer therapy and were categorized into non-statin (10,920) and statin (1,537) groups. These groups were matched using a propensity score, resulting in 2,548 non-statin and 1,274 statin users. The primary endpoints were the incidence of CIPN post-first prescription of each anticancer drug and overall survival.

**Results:**

Treatment with statins did not increase the incidence of CIPN (non-statin 27.2% vs. statin 28.4%, *P* = 0.443). Nevertheless, the incidence of CIPN was significantly high among women (non-statin 28.0% vs. statin 33.2%, *P* = 0.025). Overall survival was not impacted by statin use (hazard ratio 0.98, 95%CI: 0.83–1.16, *P* = 0.8846). Among men treated with paclitaxel, we observed an improvement in overall survival (hazard ratio: 0.72; 95% CI: 0.56–0.92; *P* = 0.0110).

**Conclusions:**

The use of statins in patients with cancer was not associated with CIPN incidence. However, in men receiving paclitaxel treatment, statins may be linked to improved overall survival. Further studies are necessary to clarify the factors influencing prognosis and CIPN severity.

## Background

Anticancer drug therapy is associated with various adverse events such as nausea, vomiting, and myelosuppression [1, 2]. However, optimal concomitant medications can mitigate these risks and potentially enhance patient survival [3]. The more frequent adverse events include gastrointestinal disturbances and sensory abnormalities such as chemotherapy-induced peripheral neuropathy (CIPN). CIPN results from the use of platinum-based, vinca-alkaloid-based, and molecular-targeted drugs [4, 5], which are commonly prescribed for colorectal, lung, and breast cancers. Repeated administration of these drugs can lead to persistent sensory abnormalities, including numbness and paresthesia, lasting beyond six months [6, 7]. While the pathogenic mechanisms of CIPN are partially understood, the efficacy of commonly used analgesics, such as NSAIDs and acetaminophen, is limited [5, 7]. Long-term sensory neuropathy, typically accompanied by motor dysfunction, remains a significant complication for cancer survivors and contributes to chronic pain, psychological dysfunction, and increased fall risk [8, 9]. Furthermore, these symptoms require alteration or cessation of treatment, highlighting the urgent need for innovative therapeutic strategies. Several pathways have been implicated in the development of CIPN; these include direct cellular damage from anticancer drugs, increased mitochondrial ROS production, and alterations in ion channel activities [10–12]. However, the underlying mechanisms remain unclear, and potential targets have been difficult to identify from pathological analyses.

Drugs typically exhibit a “main effect” targeting specific diseases and “side effects” or adverse reactions in non-target organs. Occasionally, the effect of a drug on a non-target organ may yield therapeutic benefits for other conditions [13–15]. Therefore, we hypothesized that assessing the concomitant medications might help reduce the severity of CIPN.

Statins, also known as 3-hydroxy-3-methylglutaryl coenzyme A reductase inhibitors, are dyslipidemia drugs that inhibit cholesterol synthesis in the liver. Beyond their primary pharmacological action, statins also exhibit other effects, such as anti-inflammatory effects and improvement of endothelial function, independently of their primary pharmacological action [16–19]. The effects of statins on the nervous system have also been suggested [20–22]. Activation of the transcriptional regulator Nurr1 by statins is important for neuroprotection in central neurodegenerative disorders, such as Alzheimer’s disease and Parkinson’s disease [23]. Moreover, statins exert neuroprotective effects by reducing glutamate excitotoxicity and inhibiting vascular damage in ischemic stroke [24, 25]. Although the impact of statins on the central nervous system is well known, their role in CIPN remains underexplored. We previously reported that simvastatin may be effective against CIPN in oxaliplatin-induced peripheral neuropathy (PN) based on the analysis of data retrieved from the FDA’s database of spontaneous adverse event reports, a database of self-reporting adverse events [26]. Given the widespread use of statins and their well-documented long-term safety, they may serve as viable candidates for supportive care in CIPN management. Cytotoxic anticancer agents are associated with a higher incidence of CIPN compared to antibody-based anticancer agents. While the mechanisms of neuropathy vary among different anticancer agents, the clinical symptoms are generally similar. Statins may offer potential effectiveness in managing CIPN induced by non-platinum anticancer agents.

Considering these factors, in this study, we investigated whether statins affect the risk of cytotoxic anticancer (oxaliplatin, paclitaxel, and nab-paclitaxel) induced-CIPN or compromise cancer treatment efficacy through a retrospective analysis of medical database records.

## Methods

### Study design and participants

**Fig. 1:**
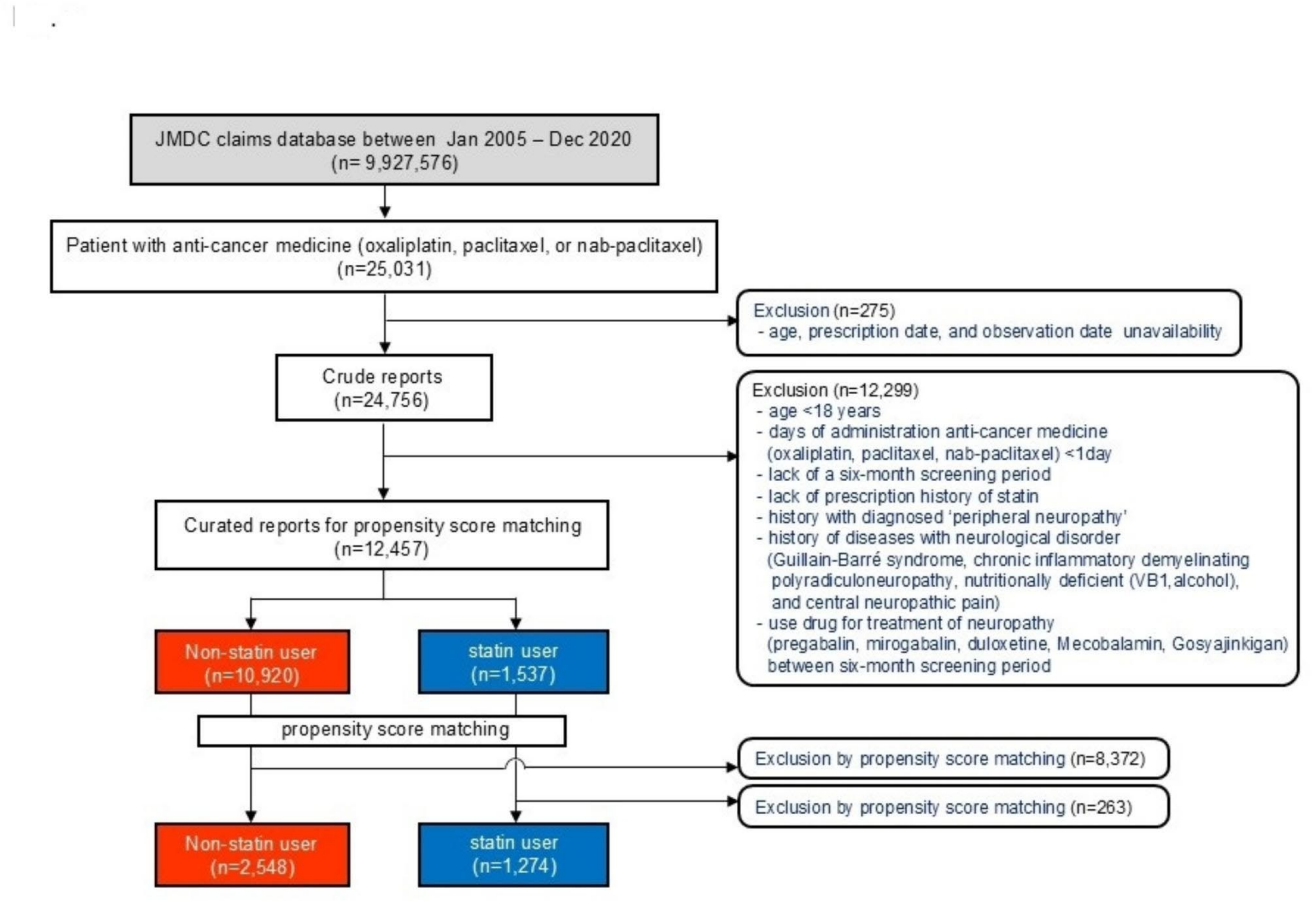
Flowchart of the curated report selection

**Fig. 2:**
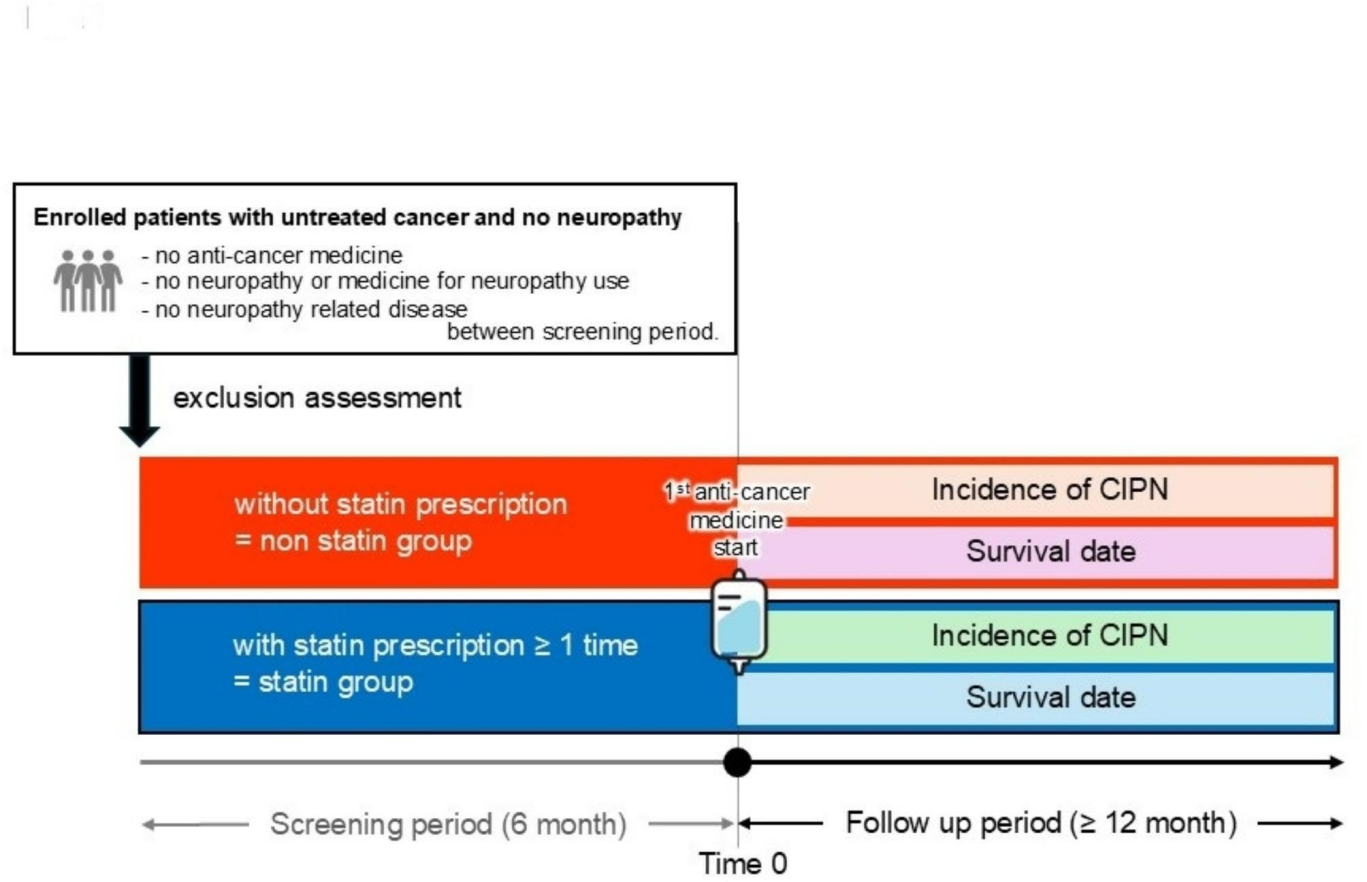
Patient selection and outcome assessment

This retrospective observational study used the Japan Medical Data Centre (JMDC) Insured Persons Database (JMDC Inc., Tokyo, Japan) [27], which contains data accumulated from January 2005 to December 2020 (Fig. 1). The entire dataset contains data through the year 2021; however, due to the necessary follow-up period for the outcomes, the final analysis was limited to data up toDecember 2020. Data from patients with cancer aged ≥ 18 years who used oxaliplatin, paclitaxel, or nab-paclitaxel were included in the analysis. We designed two periods for grouping patients: (1) a screening period (before 6 months from Time 0) to ensure comparable case backgrounds, and (2) a follow-up period (after Time 0) to analyze the development of neuropathy following anticancer drug administration. Data from patients with cancer who received oxaliplatin, paclitaxel, or nab-paclitaxel were included in the analysis. A total of 275 patients with unavailable data (age, prescription history, or observation date) were excluded from the 25,031 identified cases. To ensure a clear distinction between pre-existing neuropathy and chemotherapy-induced peripheral neuropathy (CIPN), a screening period of 6 months before chemotherapy initiation was used to exclude patients with a history of peripheral neuropathy or prior use of neuropathic pain medications. A follow-up period (≥ 1 year) after chemotherapy initiation (Time 0) was used to analyze CIPN incidence (Fig. 2). A total of 12,299 cases were excluded if they met any of the following criteria: (1) age < 18 years, (2) prior use of anticancer drugs, (3) a pre-existing diagnosis of peripheral neuropathy (identified using WHO ICD-10 codes: G62, G64, G98, M79.2, R20, and R52), or (4) prior use of neuropathic pain medications (pregabalin, mirogabalin, duloxetine, vitamin B12, and Gosyajinkigan) during the screening period. These exclusions ensured that CIPN cases identified during the follow-up period were newly developed, reducing potential confounding effects. The following neuropathy-related diseases were selected to be excluded during the screening period as patient background: Guillain–Barre syndrome, chronic inflammatory demyelinating polyradiculoneuropathy, nutritionally deficient [vitamin B12, alcohol] neuropathies, central neuropathic pain, neuropathic spinal disorders, and cauda equina neuropathy. The non-statin group was defined as patients who did not use statins during the screening or follow-up periods, while the statin group was defined as patients who were prescribed statins at least once during these periods. The statins included were atorvastatin, fluvastatin, pitavastatin, pravastatin, rosuvastatin, and simvastatin. After applying the above exclusion criteria, 12,457 patients (non-statin users: 10,920; statin users: 1,537) were included for propensity score matching. Tumor type was defined as the primary disease name that met the following criteria: (1) had an approved indication for analysis; (2) was non-metastatic; and (3) was recorded at the initiation of anticancer therapy. Baseline variables used as covariates for propensity score matching were age, sex, type of anticancer medicine, principal tumor type, hypertension, diabetes mellitus, and stroke. Propensity scores were calculated using a multivariate logistic regression model. The degree of balance was evaluated using standardized mean differences (SMD); an SMD > 0.1 indicated that an imbalance remained between the groups. The matched population (non-statin: 2,548; statin: 1,274) was used for the final analysis. No patients or members of the public were involved in the study design or data collection. Medical information was used after anonymization by JMDC Inc.

### Definition of disease and drug use

The earliest prescription date for chemotherapy administration after the screening period was defined as the first administration of the anticancer drug (Time 0). CIPN was then defined based on its first occurrence after Time 0, using the following criteria: (1) A new diagnosis of peripheral neuropathy (WHO ICD-10 codes: G62, G64, G98, M79.2, R20, R52). (2) A new prescription of neuropathic pain medication (pregabalin, mirogabalin, duloxetine, vitamin B12, or Gosyajinkigan). Without CIPN, the case with no ICD-10 code for CIPN was defined after the anticancer drug was administered. Patients with a history of peripheral neuropathy owing to diabetes or stroke were excluded from the analysis. The selection of comorbidities (diabetes, hypertension, and stroke) that may influence CIPN in patients using statins was based on ICD-10 codes. The specific codes used were as follows: diabetes (E10, E11, E12, E13, E14), hypertension (I10, I150, I151, I152, I159, O13, O100), and stroke (I630, I631, I632, I633, I634, I635, I636, I638, I639, I64, I693). The administration period in each case was defined as the number of days from the date of the first dose to the last dose. Continuous administration was defined as different first- and last-administration dates. Analgesics used during the entire period under analysis were investigated as concomitant medications. The presence of neuropathy was identified using diagnostic codes, along with the prescription of neuropathy-related medications, such as pregabalin, mirogabalin, duloxetine, vitamin B12, and Gosyajinkigan. Other analgesics were selected based on the Anatomical Therapeutic Chemical Classification System codes. Drugs were classified according to the general name of the medicine and dosage form. Those classified as cold remedies or ophthalmic remedies were excluded.

### Outcomes

The primary endpoints were the incidence and duration of CIPN with and without concomitant statins in patients receiving anticancer drugs. We also examined the (1) incidence of CIPN and the overall survival stratified by anticancer drug, cancer type, and sex, and (2) duration of chemotherapy as secondary endpoints.

### Statistical analysis

IBM SPSS Statistics for Windows, version 28.0.1.0 (IBM Corp., Armonk, NY, USA) was used for all data analysis. Primary endpoints were compared using the log-rank test (Mantel-Cox test). Comparisons between two groups for each subgroup were analyzed using the Wilcoxon rank sum test, Pearson’s chi-square test, or Fisher’s exact test. Statistical significance was set at *P* < 0.05.

## Results

We collected data for 25,031 patients with cancer who received one of three anticancer medicines—oxaliplatin, paclitaxel, or nab-paclitaxel—associated with the highest frequency of CIPN from the database (Fig. 1). The patients were divided into two groups (non-statin: 10,920; statin: 1,537) based on whether they received statin treatment. Significant differences in baseline characteristics were observed between the non-statin and statin groups, including sex (male) [non-statin: 41.3% and statin: 50.9%, *P* < 0.001], age [non-statin: 54.17 years (range 18.0–75.0) and statin: 60.77 years (range 31.2–75.0), *P* < 0.001], and stroke-related complications [non-statin: 4.1% and statin: 9.49%, *P* < 0.001] (Table 1). After propensity score matching, 3,822 patients (non-statin: 2,548; statin: 1,274) with similar baseline characteristics were included in the analysis.

**Table 1:** Baseline characteristics with or without propensity score matching

	Curated report						Propensity score match report					
	Non-statin		statin		*P* value	SMD	Non-statin		statin		*P* value	SMD
	(n = 10,920)		(n = 1,537)				(n = 2,548)		(n = 1,274)			
Sex, Male, n (%)	4,515	(41.3)	782	(50.9)	< 0.001	0.192	1,346	(52.8)	673	(52.8)	1.000	< 0.001
Age, years	54.17	(18.0–75.0)	60.77	(31.2–75.0)	< 0.001	0.757	60.06	(7.15)	60.11	(7.19)	0.835	0.007
Principal cancer, n (%)					NA	0.214					NA	0.084
Stomach	106	(0.97)	11	(0.71)			16	(0.6)	6	(0.5)		
Colorectum	464	(4.24)	70	(4.55)			105	(4.1)	55	(4.3)		
Pancreas	311	(2.84)	59	(3.83)			95	(3.7)	52	(4.1)		
Ovary	1,308	(11.9)	106	(6.89)			168	(6.6)	94	(7.4)		
Lung	820	(7.5)	142	(9.23)			212	(8.3)	120	(9.4)		
Uterus	922	(8.44)	107	(6.96)			164	(6.4)	94	(7.4)		
Breast	580	(5.31)	64	(4.16)			93	(3.6)	51	(4.0)		
Others	5,177	(47.4)	777	(50.5)			1,433	(56.2)	671	(52.7)		
Unknown	1,348	(12.3)	201	(13.0)			262	(10.3)	131	(10.3)		
Anticancer medicine, n (%)					NA	0.168					1.000	< 0.001
Oxaliplatin	4,300	(39.4)	649	(42.2)			1,104	(43.3)	552	(43.3)		
Paclitaxel	5,012	(45.9)	590	(38.3)			962	(37.8)	481	(37.8)		
nab-Paclitaxel	1,608	(14.7)	298	(19.3)			482	(18.9)	241	(18.9)		
Complication, n (%)												
Diabetes mellitus	6,621	(60.6)	1,289	(83.8)	NA	0.564	2,182	(85.6)	1,091	(85.6)	1.000	< 0.001
Hypertension	2,836	(26.0)	1,001	(65.1)	NA	0.861	1,596	(62.6)	798	(62.6)	1.000	< 0.001
Stroke	451	(4.1)	146	(9.49)	< 0.001	0.222	140	(5.5)	70	(5.5)	1.000	< 0.001

Oxaliplatin was the most commonly used anticancer medicine among patients, followed by paclitaxel and nab-paclitaxel. Diabetes (85.6%) was the most common comorbidity among patients, whereas stroke (5.5%) was the least common. In propensity score-matched cases, the incidence of CIPN was similar in both groups [non-statin: 694 (27.2%); statin: 362 (28.4%), *P* = 0.443]. In cases of CIPN, 645 non-statin users had both a diagnostic code and neuropathic pain medication prescriptions, while 49 cases (7%) had a diagnosis alone. Among statin users, 343 cases had both a diagnosis and medication, while 19 cases (5%) had only a diagnosis. All patients in the diagnosis-only group were taking other analgesics that were not classified as neuropathic pain medications (data not shown). However, the incidence of CIPN was significantly higher in women receiving statins [non-statin: 337 (28.0%); statin: 200 (33.2%), *P* = 0.025] (Table 2). Evaluation of the incidence of CIPN for each anticancer drug demonstrated no significant changes overall.

**Table 2:** Changes in the incidence of CIPN and duration of chemotherapy

	Propensity score matching report				
	Non-statin (*n* = 2,548)		statin (*n* = 1,274)		*P* value
Incidence of PN, n (%)	Events		Events		
Total	694	(27.2)	362	(28.4)	0.443
Male	357	(26.5)	162	(24.0)	0.257
Female	337	(28.0)	200	(33.2)	0.025
Anticancer medicine					
Oxaliplatin	258	(23.3)	131	(23.7)	0.902
Male	191	(24.8)	91	(23.6)	0.716
Female	67	(19.9)	40	(23.8)	0.355
Paclitaxel	312	(32.4)	166	(34.5)	0.441
Male	70	(32.1)	25	(22.9)	0.094
Female	242	(32.5)	141	(37.9)	0.082
nab-Paclitaxel	124	(25.7)	65	(26.9)	0.720
Male	96	(26.6)	46	(25.5)	0.836
Female	28	(22.9)	19	(31.1)	0.282
Principal cancer					
Stomach	4	(25.0)	0	(0)	0.541
Colorectum	23	(21.9)	11	(20.0)	0.841
Pancreas	19	(20.0)	23	(44.2)	0.002
Ovary	58	(34.5)	34	(36.1)	0.789
Lung	55	(25.9)	28	(23.3)	0.692
Uterus	50	(30.4)	35	(37.2)	0.274
Breast	29	(31.1)	15	(29.4)	0.852
Others	399	(27.8)	191	(28.4)	0.795
Unknown	57	(21.7)	25	(19.0)	0.599
Duration of treatment days					
Total	164.90		163.60		0.862
Male	173.19		165.91		0.465
Female	155.63		161.12		0.599
Anticancer medicine					
Oxaliplatin	183.79		161.93		0.054
Male	196.07		169.22		0.058
Female	155.71		145.25		0.568
Paclitaxel	145.41		154.14		0.426
Male	114.76		119.27		0.802
Female	154.39		164.36		0.448
nab-Paclitaxel	160.57		186.58		0.128
Male	159.76		187.07		0.136
Female	162.97		185.11		0.586
Principal cancer					
Stomach	99.25		131.17		0.693
Colorectum	213.18		186.16		0.536
Pancreas	227.99		230.67		0.960
Ovary	166.34		158.72		0.768
Lung	171.55		150.81		0.340
Uterus	159.62		152.91		0.781
Breast	141.95		132.53		0.807
Others	165.24		171.41		0.540
Unknown	129.99		124.44		0.695

In the case of paclitaxel, a trend toward a reduced incidence of CIPN was observed among men receiving statins compared with that in non-statin users [non-statin: 70 (32.1%); statin: 25 (22.9%), *P* = 0.094]. In contrast, a trend toward a higher incidence of CIPN was observed in women receiving statins compared with that in non-statin users [non-statin: 242 (32.5%); statin: 141 (37.9%), *P* = 0.082]. Furthermore, statin treatment significantly increased the incidence of CIPN in patients with pancreatic cancer [non-statin: 19 (20.0%); statin: 23 (44.2%), *P* = 0.002].

**Fig. 3:**
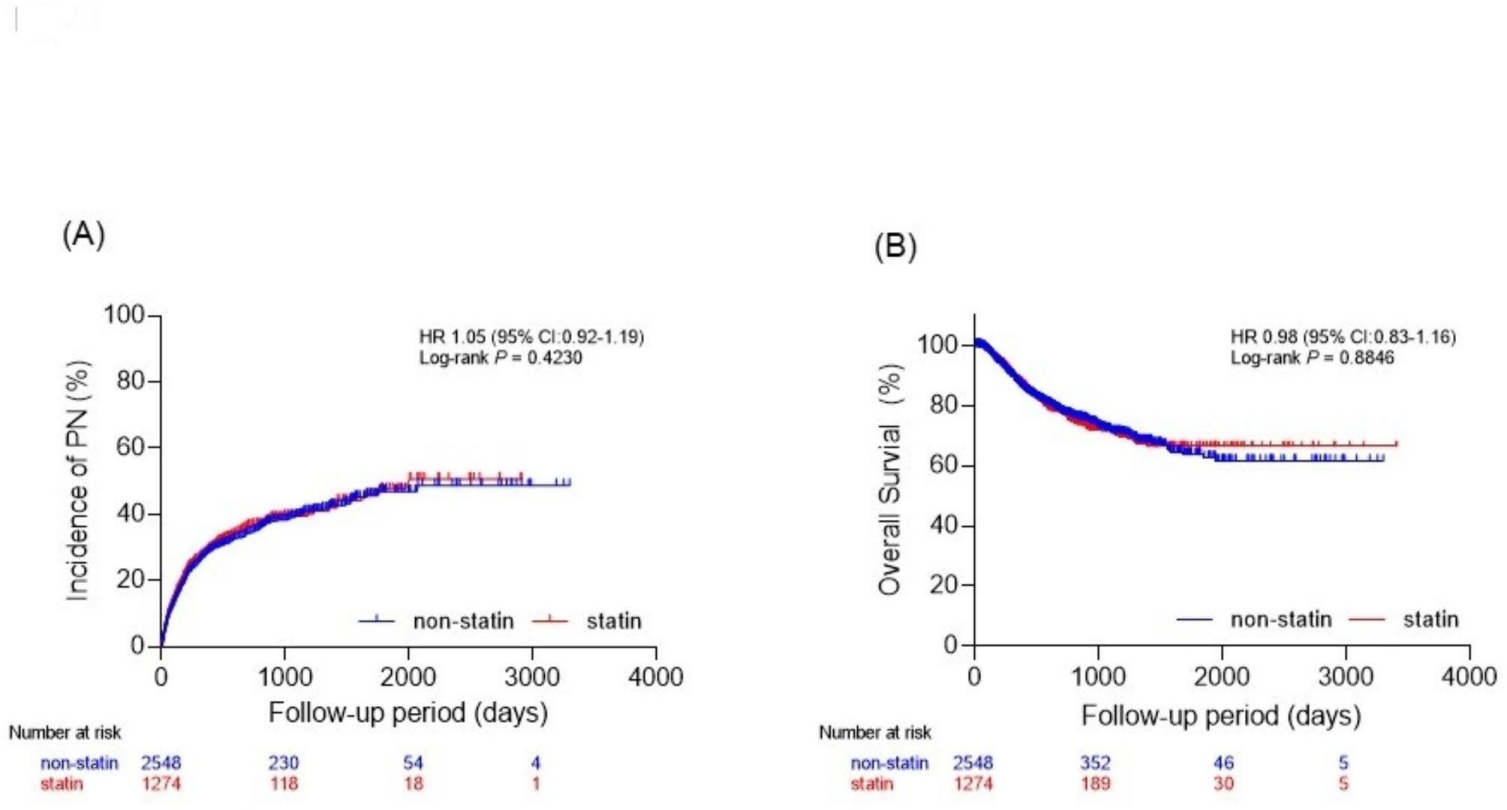
Changes in the incidence of peripheral neuropathy and overall survival after the first chemotherapy (**A**). Accumulated incidence of CIPN. (**B**). Overall survival of both groups

**Fig. 4:**
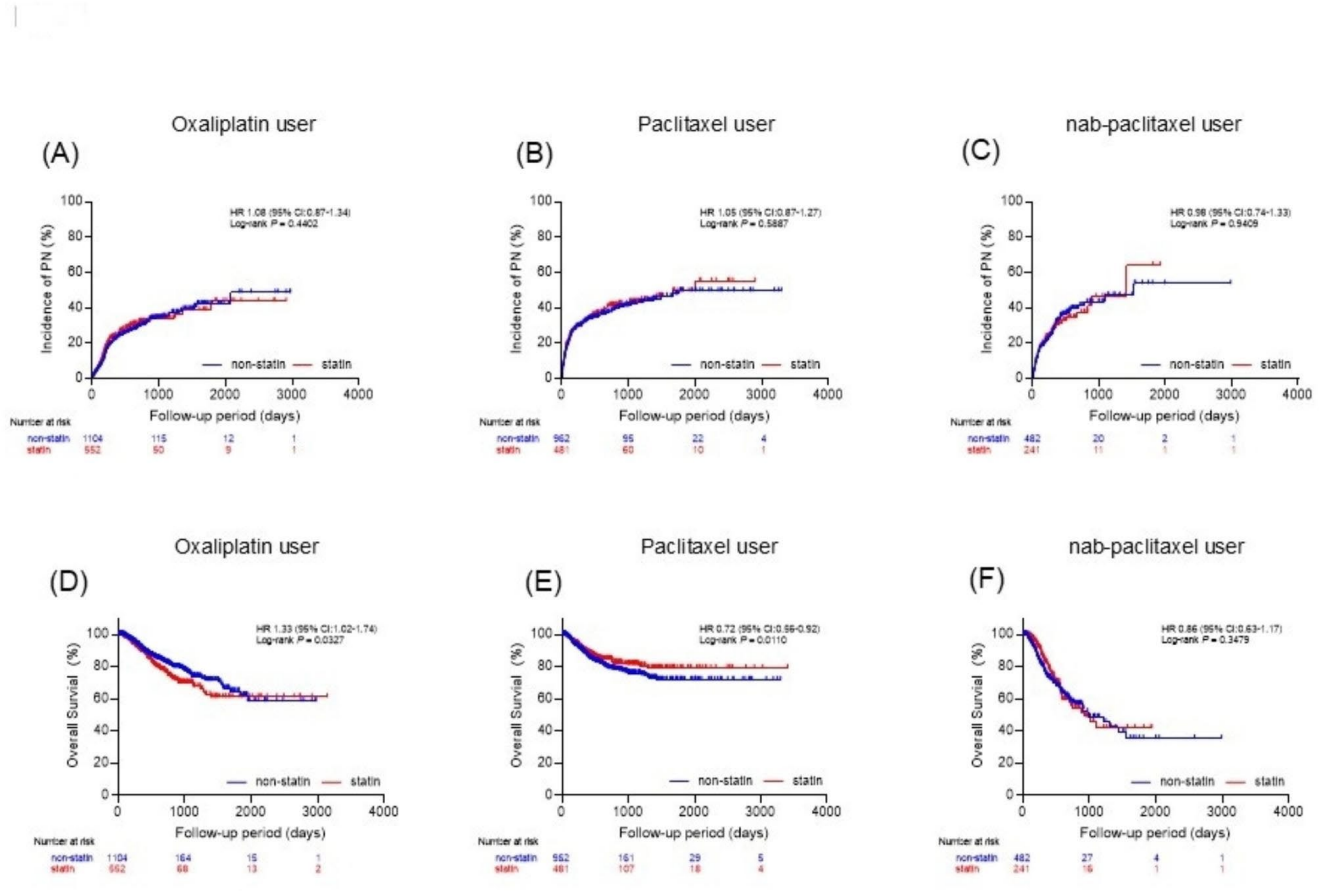
Secondary outcome for each anticancer medicine (**A-C**) Accumulated incidence of each anticancer medicine. (**D-F**) Overall survival of patients with each anticancer medicine

The cumulative incidence of CIPN during the follow-up period was not significantly different between the two groups [hazard ratio (HR): 1.05; 95% confidence interval (CI): 0.92–1.19; Log-rank *P* = 0.4230; Fig. 3A]. The cumulative incidence of CIPN was also similar between the two groups when analyzed by type of anticancer drug (Fig. 4A–C). The average duration of anticancer treatment was similar (non-statin: 164.90 days; statin: 163.60 days; *P* = 0.862). These findings were consistent when analyzed by sex and principal cancer type.

The analysis of overall survival among all cases revealed no statistically significant difference between the non-statin and statin groups [HR: 0.98; 95% CI: 0.83–1.16; Log-rank *P* = 0.8846; Fig. 3B]. However, when analyzed according to the anticancer drug used, results varied. Treatment with oxaliplatin was associated with worse overall survival in the statin group [HR: 1.33; 95% CI: 1.02–1.74; Log-rank *P* = 0.0327; Fig. 4D], while paclitaxel was associated with improved overall survival [HR: 0.72; 95% CI: 0.56–0.92; Log-rank *P* = 0.0110; Fig. 4E]. Patients treated with nab-paclitaxel showed no significant effect of statin use on survival [HR: 0.86; 95% CI: 0.63–1.17; Log-rank *P* = 0.3479; Fig. 4F].

## Discussion

Drug–drug interactions can be both beneficial and detrimental for patients [28, 29]. Specifically, statins exert neuroprotective effects [30] but have also been associated with a risk of neuropathy [31]. Given that CIPN is a common side effect of many anticancer therapies, the potential interaction between anticancer medicines and statins may influence the development and severity of CIPN. Understanding how statins interact with these therapies is critical for managing neuropathy in patients with cancer. Although CIPN can occur with various classes of anticancer medicines, CIPN is more frequently observed with cytotoxic agents such as platinum compounds, taxanes, and vinca alkaloids, which are used in a large proportion of patients with cancer. In this study, we analyzed CIPN incidence and survival in patients treated with oxaliplatin, paclitaxel, and nab-paclitaxel, which are commonly used anticancer drugs. The incidence of CIPN is reportedly 60–80%; however, the incidence is particularly unclear in Japan and varies from report to report [6]. We examined the incidence of CIPN with a follow-up period of at least one year using the JMDC database. We used this database because its linkage to insurance information enables continuous patient follow-up even when patients change medical institutions, allowing us to capture more comprehensive and long-term data.

Examination of the incidence of CIPN with the first administration of anticancer medicines indicated that the rate of neuropathy was approximately 30%, and the cumulative incidence was approximately 60%, with no significant difference in early-stage rates between those receiving statins and those not. The onset period for CIPN is estimated to range from a few days to a few months. In this study, the earliest report of CIPN was on day 3 post-anticancer drug administration, with or without statin combination, and the time until 50% of all patients developed CIPN was 252 days for non-statins and 243 days for statins. These results indicated that half of the patients developed CIPN within one year after anticancer drug administration. In the present study, the statin group includes patients who were prescribed statins after the administration of anticancer drugs. In more than 90% of cases, patients had been using statins prior to chemotherapy. Given that most of the data analyzed in this study are from patients who were on statins before the initiation of anticancer therapy, our results suggest that the effect of statin pre-administration during the first anticancer treatment has been assessed. Additionally, we have demonstrated in animal studies the efficacy of statins after the development of CIPN, showing that statins can suppress CIPN even when administered after anticancer therapy [32]. Therefore, statins may be less effective in reducing the initial occurrence of CIPN but might serve as a long-term recovery aid. More specific studies are needed to clarify these details by collecting cases in which statins were administered from the onset of the disease and evaluating their efficacy.

CIPN is generally regarded as a limiting factor in anticancer drug administration, often leading to dose reductions or early discontinuation [33], which may adversely affect patient outcomes [34]. Our hypothesis was that if statins mitigate CIPN, they might help maintain the full chemotherapy dose, contributing to improved OS. However, as we found no significant difference in CIPN incidence between the groups, the observed improvement in OS with paclitaxel suggests that statins may influence survival through mechanisms unrelated to CIPN mitigation. Few studies have directly examined the relationship between CIPN and survival. Shah et al. reported a 5-year survival rate of 55.2% in CIPN patients compared to 36.1% in non-CIPN patients [35]. Although the association between CIPN and survival remains uncertain, statins have been linked to reduced cancer recurrence and improved survival in patients with breast cancer and in those receiving immune checkpoint inhibitors [36, 37]. Additionally, chemotherapy dose intensity has been positively correlated with survival outcomes [38–40]. These findings suggest that the survival benefit observed in our study may be due to either statins mitigating neuropathy, allowing continued chemotherapy administration, or a broader protective effect of statins independent of CIPN.

CIPN has long-term persistent symptoms, with approximately 30% of patients complaining of symptoms persisting for more than six months. Additionally, a time lag between symptom onset, diagnosis, and intervention is expected. Consequently, the number of patients is expected to be slightly lower than what is observed in actual clinical practice; however, the number of potential patients is likely to be large, leading to a high cumulative incidence rate. To prevent severe cases, early detection is necessary; therefore, even minor symptoms should be monitored regularly for up to one year after administration, and treatment plans for anticancer agents may need to be revised as needed.

Assessing the incidence of CIPN by sex revealed that the risk of neuropathy was higher in women receiving statins than in men, and the incidence was also higher with each anticancer drug. In contrast, it did not significantly affect the incidence of CIPN, and improved survival was observed in men receiving paclitaxel and statins. Sex differences reportedly exist in lipid metabolism and pain sensitivity [41–43]. The anticancer drugs analyzed in the present study are also used in cancers that predominantly affect women. Chemotherapy-induced early menopause can influence lipid metabolism and may contribute to sex differences in treatment outcomes. To explore this, we analyzed ovarian, uterine, and breast cancer cases, all of which were exclusively female patients. The typical age of menopause is between 40 and 45 years, and the adolescent and young adult cancer population is defined as ages 15–39 [44]. However, no cases of ovarian or breast cancer included women under 40 years old who had experienced early menopause. In uterine cancer, such cases accounted for less than 3%. Because of this limited sample size, a meaningful comparison of early menopause cases was not feasible. Additionally, the dataset does not contain direct information on menopause status, limiting our ability to assess its impact. The expression of molecules related to pain sensation differs between men and women. Furthermore, both lipid metabolism and pain sensitivity are regulated by the sex hormone estrogen [45, 46]. Changes in estrogen levels may contribute to observed sex differences. However, as most female patients in this study were middle-aged or older, they were likely postmenopausal, and any estrogen-related effects remain unclear. To further investigate the role of sex differences, future studies should incorporate a younger population with detailed menopause status data. Many patients on statins also have comorbidities like diabetes and stroke, which are independent risk factors for CIPN. Therefore, neuropathy may occur at a high frequency in patients currently using statins. Statins reportedly cause neuropathy as an adverse reaction [47]; however, we did not confirm this observation in patients treated with anticancer agents. In our animal model study, we reported that high doses of statins had a suppressive effect on oxaliplatin- and paclitaxel-induced neuropathy [32]. In a randomized, double-blind, placebo-controlled trial, nerve damage in patients with type 2 diabetes was reduced by statin treatment [17]. Simvastatin reportedly has direct protective effects, such as improving vincristine-induced PN in a rat model [48]. The absence of an increase in CIPN in the present study could be attributed to the suppression of lifestyle-related disease exacerbations and direct neuroprotective effects of statin treatment.

After completing treatment, some patients transition to local care, complicating long-term follow-up and accurate CIPN monitoring. Thus, evaluating the actual status of CIPN occurrence using only information from a single medical institution has been difficult. We used a medical database to determine the occurrence of CIPN in patients with cancer over time and to show, for the first time, the risk factors for CIPN due to drug interactions. However, we acknowledge several limitations in the study. The first limitation pertains to the race of the analyzed database. The database used is based on cases treated by Japanese insurance, which may include some patients with foreign backgrounds; however, the majority of cases are Japanese. The detailed analysis data differed from those of previous studies, which were mainly based on data from other countries [26]. This might be attributed to differences in the doses of approved drugs as well as race. Our data would be useful for Japanese patients and patients from other countries with similar backgrounds; however, more extensive population data analysis is needed to examine the generalizability of the data worldwide. The second limitation was the CIPN severity comparison. The occurrence of CIPN was defined based on diagnostic codes and the use of neuropathic pain medications. In the analysis, more than 90% of patients in all groups had both diagnostic codes and neuropathic pain medications prescribed. The remaining 10% had diagnostic codes alone, and an analysis of concomitant medications revealed that these patients were prescribed opioids and non-steroidal anti-inflammatory drugs. These findings suggest that the majority (90%) of CIPN cases analyzed were grade 2 or higher. However, this definition does not account for mild symptoms or cases where no therapeutic intervention was recorded. Furthermore, the dataset did not include information on neuropathy severity, limiting the ability to assess whether statin administration influences CIPN severity. A sensitivity analysis using neuropathic pain medication prescriptions could further refine these findings, and we aim to address this in future research. Prospective observational studies at multiple institutions using a uniform evaluation system are required to clarify this impact. The third limitation is the assessment of the risk of CIPN exacerbation by other drugs. Patients with and without statins may have different backgrounds regarding concomitant medications, physical size, and comorbid lifestyle-related diseases. In the present study, to minimize such differences, we used data matched for background factors, including age, sex, comorbidities, anticancer drugs used, and types of cancer treated. Because the medication regimens reflect the medical situation in a single country, significant differences between patients are unlikely; however, as more concomitant medications are used, drug interactions become more common. The average age of the patients in this study was 60 years, but the number of medications used increases with age. Polypharmacy, which increases with age, is a worldwide problem, and more than half of patients over the age of 60 may have polypharmacy. The antidiabetic drugs metformin and alogliptin and the dyslipidemic drug ω-3 fatty acids reportedly suppress various CIPNs in rodents and humans [49–51]. Although these effects are positive for CIPN, these benefits may be lost and worsen the physical condition of the patients, as those with cancer are generally treated with more drugs. Predicting and estimating all drug interactions is difficult; therefore, our results may not represent the pure effects of statins alone. Although the pharmacological effects have been studied in animal models, pure statin-only effects in humans should be studied in patients with cancer without dyslipidemia or other influencing factors.

## Conclusions

The use of statins during anticancer treatment may require closer monitoring in women, whereas long-term benefits were suggested for men receiving paclitaxel. Supportive care of patients with cancer for acute side effects is well established, whereas measures for prolonged and refractory side effects are lacking. Survival rates of patients with cancer are improving year by year, and maintaining a healthy life before and after cancer also warrants attention. Intervention is required at the point of managing adverse events caused by drug interactions before a therapeutic agent can be established. During anticancer drug treatment, patients face multiple challenges, including physical decline and potential drug interactions. Our findings do not support a role for statins in CIPN prevention; however, their potential impact on long-term outcomes warrants further investigation. Future research should explore the influence of statins on symptom severity and long-term prognosis to optimize pharmacological therapy for cancer patients.

## Data Availability

The original contributions presented in the study are included in the article; further inquiries can be directed to the corresponding author.

## Abbreviations

CIPN: Chemotherapy-induced peripheral neuropathy
JMDC: Japan Medical Data Centre
NeuSTART: Neuroprotection with statin therapy for acute recovery trial
PN: Peripheral neuropathy
SMD: Standardized mean differences
